# Transient spinal cord dysfunction after surgery for intraspinal tumors: A case report

**DOI:** 10.1097/MD.0000000000035970

**Published:** 2023-11-10

**Authors:** Zhikang Tian, Qingwei Li, Sheng Gao, Chunyang Meng

**Affiliations:** a Jining Medical University, Jining, China; b Affiliated Hospital of Jining Medical University, Jining, China.

**Keywords:** intraspinal tumor, meningioma, temporary dysfunction of the spinal cord

## Abstract

**Rationale:**

Limb dysfunction is not uncommon clinically after intramural tumor surgery. However, there are no relevant literature reports on the recovery of unilateral motor function caused by spinal cord dysfunction after short-term observation and treatment. The report of such cases is of great value for improving the cognition of postoperative complications of meningioma reducing misdiagnosis and providing reference for clinical treatment.

**Patient concerns:**

A 73-year-old female patient with numbness and weakness in both lower limbs accompanied by unstable walking for 2 months. Combined with imaging data and postoperative pathological diagnosis, it was diagnosed as thoracic spinal meningioma. The patient experienced transient unilateral limb dysfunction after surgery.

**Diagnoses:**

Magnetic resonance imaging and its enhanced magnetic resonance imaging suggest a space occupying lesion on the left side of the spinal canal at the level of the thoracic 3 to 4 vertebral body, possibly a meningioma. The postoperative pathology was grade I meningioma.

**Intervention:**

Administer 10 mL of dexamethasone, 1 g of methylprednisolone, and 250 mL of mannitol for treatment.

**Outcomes:**

After 3 hours, the patient’s muscle strength gradually recovered, and after 12 hours, it was better than the preoperative level.

**Conclusion:**

Spinal cord dysfunction may occur after surgery for intraspinal meningioma in the upper thoracic spine. Unlike spinal cord dysfunction caused by spinal cord injury, this dysfunction is short-term and transient. The use of hormones and diuretics is a feasible solution that can quickly restore patient limb function.

## 1. Introduction

Intraspinal tumor is one of the common central nervous system tumors. Relevant literature data show that intradural nervous system tumors account for about 3.1% of primary central nervous system tumors,^[1]^ and benign meningiomas are especially common, accounting for 25% to 30% of all intradural extramedullary spinal tumors.^[2]^ Meningioma originates from the spinal meninges and usually occurs in the thoracic vertebrae of women over 60 years old. Clinical symptoms do not have special characteristics, and are often manifested as compression symptoms such as sensory disorders and motor weakness. Complete surgical resection of the pathological tissue within 12 months of symptom occurrence can often achieve good results. Postoperative complications include but are not limited to epidural blood, cerebrospinal fluid leakage, superficial wound infection, etc.^[3]^ Nevertheless, the cases of transient spinal dysfunction after meningioma resection are rarely reported, and the cases mainly characterized by unilateral limb movement disorders are extremely rare. This case has expanded the understanding of postoperative complications of intraspinal tumors, and our rapid and effective treatment plan for this case also provides an important reference for the treatment of such complications, so it is reported below.

## 2. Case report

The patient, a 73-year-old female, presented with numbness and weakness in both lower limbs and walking instability for 2 months. She had undergone “thyroidectomy” 27 years ago and “knee arthroplasty” 3 years ago.

**Figure 1.**
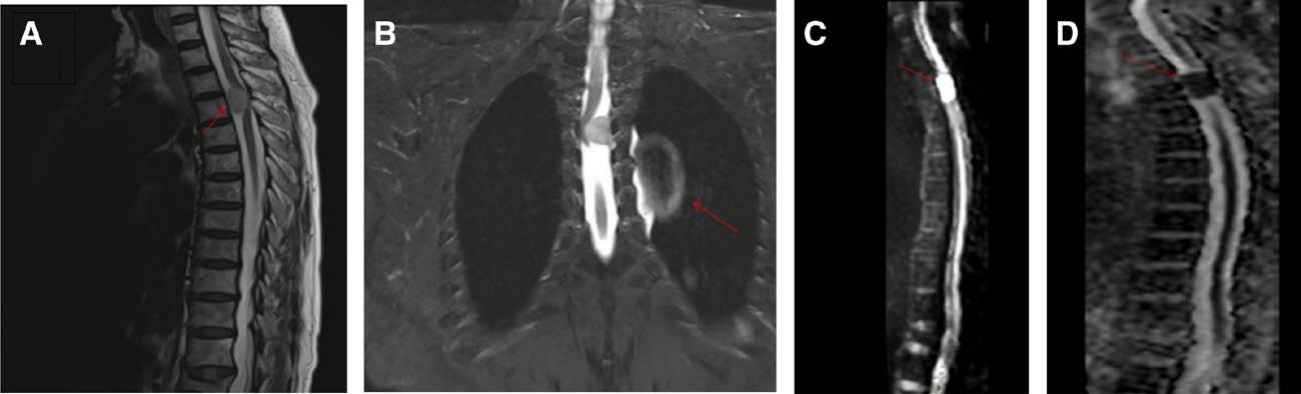
Preoperative imaging: (A) preoperative sagittal MRI; (B) preoperative coronal MRI; (C) preoperative sagittal enhanced MRI; (D) preoperative enhanced MRI. Soft tissue signal nodular shadow was seen on the left side of the spinal canal at the level of the thoracic 3 to 4 vertebral body, showing T1WI iso-signal, DWI high signal, ADC low signal, size about 14 mm × 20 mm × 12 mm, adjacent to the spinal cord compression shifted to the right, the lesion localized. The lesion appeared to be connected to the wide base of the left dura mater, and showed significant uniform enhancement after enhancement. The diagnosis is a left-sided occupying lesion at the level of the thoracic 3 to 4 vertebral body: suggesting a possible spinal meningioma. MRI = magnetic resonance imaging.

Physical examination: abdominal wall below the umbilical sensation decreased, perineal area shallow sensation decreased; right hip flexion, knee flexion, ankle flexion, and great toe dorso-extension strength level 3; left hip flexion, great toe dorso-extension strength level 3-, ankle flexion and great toe dorso-extension strength level 2+. The muscle tone of both lower limbs was high, and the anal sphincter tone was decreased. Double biceps reflex (+), triceps reflex (+), radii membrane reflex (+), bilateral Hoffmann sign (−); Slow abdominal reflex, knee reflex (+++), tendon reflex (+++), patellar clonus (−), ankle clonus (−); Pathological signs: bilateral Babinski sign (−). Thoracic magnetic resonance imaging (MRI) and enhanced MRI revealed left-sided space occupying lesions in the horizontal spinal canal of the thoracic 3-4 vertebrae: suggestive of meningioma (Fig. 1).

**Figure 2.**
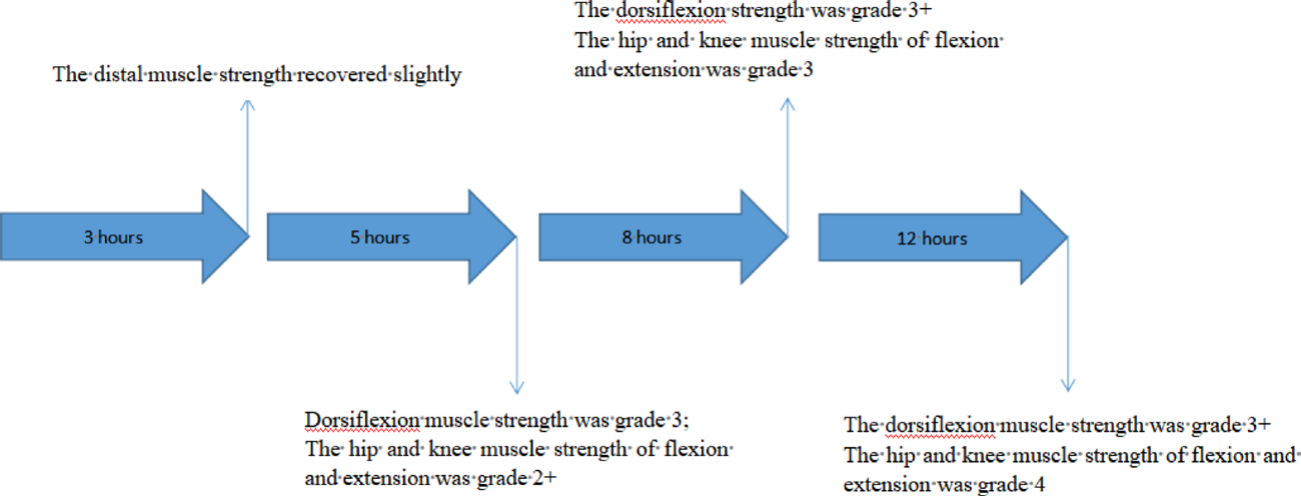
Patient muscle strength recovery time and changes in muscle strength.

**Figure 3.**
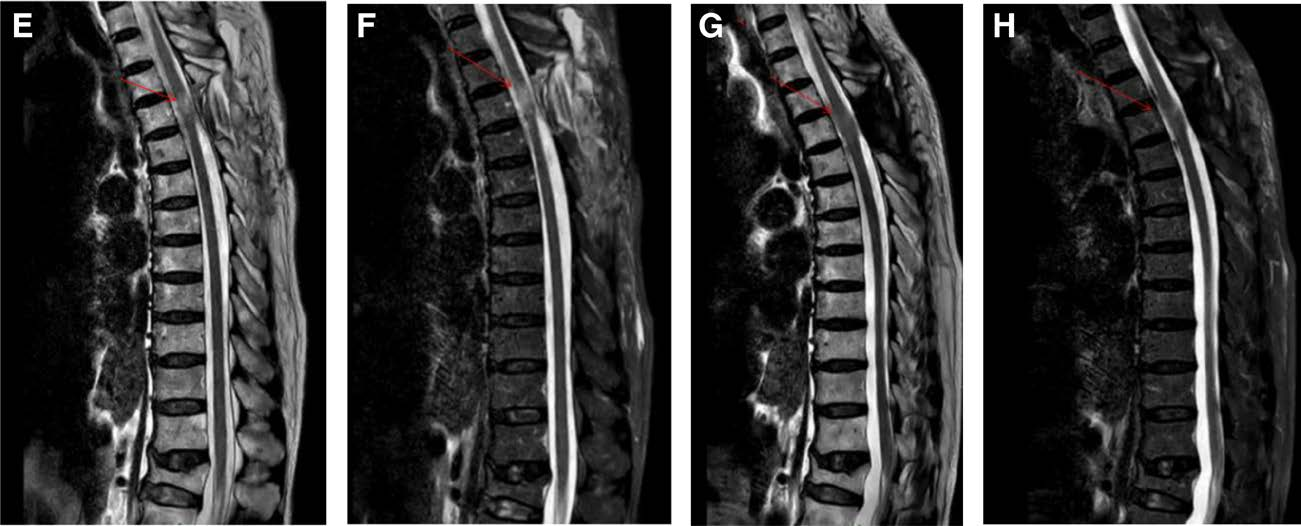
Postoperative imaging date: (E and F) Postoperative changes in sagittal MRI of the thoracic 3 to 5 vertebral canal after removal of the drainage tube, relief of spinal cord compression at the level of the thoracic 4 vertebral body, and spinal cord degeneration; (G and H) sagittal MRI at 2 months after surgery: Postoperative changes in the posterior spinal canal of the thoracic 3 to 5 vertebrae, reduced swelling of surrounding soft tissue compared to before, relieved compression of the spinal cord at the level of the thoracic 4 vertebrae, and spinal cord degeneration. MRI = magnetic resonance imaging.

**Figure 4.**
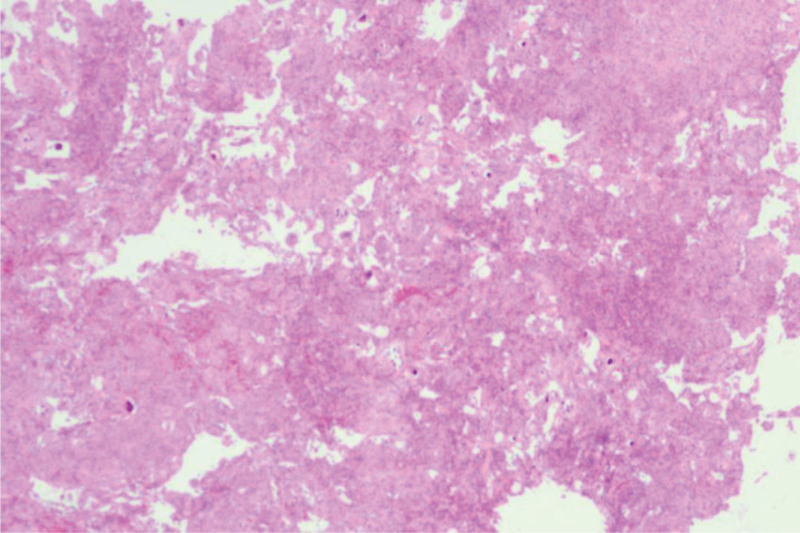
Postoperative pathology: meningeal epithelial cells were long spindle-shaped, intertwined, and swirled arrangement, with unclear borders between tumor cells, and visible sand granules. The histological diagnosis was considered spinal meningioma (WHO grade 1). Immunohistochemistry: tumor cells EMA (+), PR (+), SSTR-2 (+), H3K27me3 (+), S-100 (+), GFAP (−), MUC4 (+), abd Ki-67 (+, <2%).

During the operation, the thoracic 3/4 spinous process and lamina were removed, the dura mater was completely exposed, the dura mater was cut under microscope, the nerve dissection was explored and separated, the tumor was completely removed, the dura mater was repaired with 5-0 absorbable continuous mattress suture and artificial dura mater, and there was no cerebrospinal fluid leakage. The tissue was sutured tightly and tension-free.^[3]^ About 30 minutes after surgery, the patient was awake, and physical examination revealed immobility of the left lower limb. Physical examination showed grade 0 muscle strength in all muscle groups, sensation was present, Babinski sign (−), and knee and ankle reflexes were absent. After communicating with the patient’s family, the muscle strength of the unilateral limb decreased, and hematoma formation was not considered for the time being. About 3 hours, the muscle strength of the left lower limb recovered and the sensation returned to the preoperative level (Fig. 2). Dexamethasone 10 mg was given again, and the muscle strength of the left lower limb recovered significantly about 12 hours after the operation. Postoperative MRI showed postoperative changes of the intraspinal space occupying in the posterior thoracic vertebra 3 to 5, relief of spinal cord compression at the level of thoracic vertebra 4, and spinal cord degeneration I (Fig. 3). Postoperative pathology: the histological diagnosis was meningioma (WHO grade 1). Immunohistochemical: tumor cell EMA (+), PR (+), SSTR-2 (+), H3K27me3 (+), S-100 (+), GFAP (−), MUC4 (+), Ki-67 (+, <2%) (Fig. 4). The patient was discharged home 9 days after surgery and was able to walk unaided. Reexamination of MRI at 2 months after operation showed that the posterior spinal canal occupying lesion of thoracic vertebra 3 to 5 was changed after operation, the swelling of surrounding soft tissue was relieved, the compression of spinal cord at the level of thoracic vertebra 4 was relieved, and spinal cord degeneration was observed (Fig. 3).

## 3. Discussion

Spinal meningiomas are mostly benign tumors. For patients with severe symptoms, surgical treatment is an effective method, which can not only effectively remove the space-occupying lesions, but also take pathological tissues to clarify the nature of the tumor and effectively improve the quality of life of patients.^[3]^ Studies have shown that the vast majority (90%) of spinal meningiomas are intradural, 5% are both intradural and epidural, and 5% are completely epidural. The majority of meningiomas were lateral to the spinal cord (68%), while 18% were posterior to the spinal cord and 15% anterior to the spinal cord.^[4]^ In this case, the spinal meningioma was located in the dura and extramedullary, which had seriously compressed the spinal cord. The patient had numbness and weakness of both lower limbs, walking instability, and other symptoms, which seriously affected the quality of life.

After complete resection of the meningioma, the patient had transient limitation of movement of the left lower limb after waking up. Physical examination showed that Babinski sign was negative and autonomic nerve reflex was not evoked. The muscle strength of the lower limbs improved rapidly within 12 hours after intravenous administration of methylprednisolone sodium succinate 1 g and mannitol 250 mL.

It is speculated that there may be the following reasons: (1) Ischemia-reperfusion injury: the patient had severe spinal cord compression and spinal cord deviation before operation. After tumor resection, the blood flow and reperfusion of the spinal cord after ischemia resulted in transient spinal cord injury. (2) Drift injury of the spinal cord, similar to whiplash injury and spinal cord concussion, occurs with slight fluctuation after spinal cord compression is relieved. (3) The phenomenon of sensory-motor dissociation refers to the loss of motor function and the presence of sensory part, resulting in transient sensory-motor dissociation. The sensory-motor dissociation occurs after spinal cord injury and compression relief.

Spinal cord dysfunction often occurs in patients with spinal cord injury,^[5]^ mainly because spinal cord injury affects corticospinal conduction, leading to lower limb dysfunction.^[6]^ It is worth noting that high spinal cord injury above the 6th thoracic vertebra may lead to abnormal autonomic reflexes, resulting in serious complications such as cardiac arrest and cerebrovascular hemorrhage, so it is necessary to be vigilant in clinical work.^[7]^ However, this patient’s postoperative transient spinal cord dysfunction, which rapidly improved and recovered within 12 hours after treatment with glucocorticoids and diuretics, is rare in previous clinical work. This suggests that we should carry out detailed evaluation of imaging examinations before surgery and formulate as minimally invasive surgical approach as possible. Secondly, preoperative communication should be done well, even if there is no spinal cord injury during the operation; transient spinal cord dysfunction cannot be ruled out.

Due to the transient nature of this dysfunction, effective auxiliary examinations have not been conducted to clarify the pathogenesis. It can only indicate the possibility of such complications. I hope there will be further research reports in the future.

## 4. Conclusions

Surgical removal of meningioma can lead to complications such as epidural hemorrhage, cerebrospinal fluid leakage, and superficial wound infection. High thoracic spinal meningioma may also result in spinal cord dysfunction after complete resection of the meningioma. This spinal cord dysfunction is temporary, transient, and mainly manifested as unilateral limb dysfunction. In addition, the use of diuretics combined with hormone therapy is expected to rapidly improve spinal cord function in such patients.

## Acknowledgments

The authors would like to thank our department colleagues and these patients for their dedication, and those patients or their next of kin had signed the informed consent forms.

## Author contributions

**Project administration:** Chunyang Meng.

**Supervision:** Qingwei Li.

**Writing—original draft:** Zhikang Tian.

**Writing—review & editing:** Sheng Gao, Chunyang Meng.
